# Durability of an Epoxy Resin and Its Carbon Fiber- Reinforced Polymer Composite upon Immersion in Water, Acidic, and Alkaline Solutions

**DOI:** 10.3390/polym12030614

**Published:** 2020-03-07

**Authors:** Arya Uthaman, Guijun Xian, Sabu Thomas, Yunjia Wang, Qiang Zheng, Xiaoling Liu

**Affiliations:** 1 School of Civil Engineering, Harbin Institute of Technology, Harbin 150090, China; aryauthaman@yahoo.in; 2 International and Inter University Centre for Nanoscience and Nanotechnology (IIUCNN), Mahatma Gandhi University, Kerala 686560, India; sabuthomas@mgu.ac.in; 3 Shengli Oilfield Company, China Petroleum & Chemical Corporation (SINOPEC), Dongying 257100, China; wangyunjia.slyt@sinopec.com (Y.W.); zhengqiangupc@126.com (Q.Z.); liuxiaoling133.slyt@sinopec.com (X.L.)

**Keywords:** durability, aging conditions, carbon fiber-reinforced polymer (CFRP), glass transition temperature (T_g_), service life prediction, degradation

## Abstract

The usage of polymer composites in various engineering fields has increased. However, the long-term service performance of such materials under aggressive conditions is still poorly understood, which limits the development of safe and economically effective designs. In this study, the aging of an epoxy resin and its carbon fiber-reinforced polymer (CFRP) composites upon immersion in water, acidic, and alkaline solutions was evaluated at different temperatures. The service life of the CFRP composites under various conditions could be predicted by the Arrhenius theory. The thermal and mechanical analysis results indicated that the CFRP composites were more vulnerable to HCl owing to the higher moisture absorption and diffusion of HCl into their cracks. The scanning electron microscopy results showed that the polymer matrix was damaged and degraded. Therefore, to allow long-term application, CFRP composites must be protected from acidic environments.

## 1. Introduction

Fiber-reinforced polymer (FRP) composite materials have recently attracted significant interest in many fields, such as civil engineering and aerospace, owing to their low density, spectacular mechanical strength [1], low weight, anticipated long service-life, and resistance to corrosion [2,3,4]. FRPs are most widely used in civil infrastructure applications, such as reclamation via external bonding, in outdoor environments with concrete, for renewing various types of concrete, and in steel structures [5,6]. FRP has many advantages over steel, such as its low weight, simple installation process, and high corrosion resistance [7,8]. Carbon fiber-reinforced polymers (CFRPs) are also receiving increasing interest due to their emerging role in strengthening structural elements in construction [9,10]. To improve the toughness and strength of fibers, thermosetting polymers (epoxy, polyester, phenolic, and polyimide resins) and thermoplastics (polypropylene, and poly methyl methacrylate) have been broadly considered [2,11]. The resin matrix plays a vital role in composite materials as it transfers the load between the fibers and composite laminates in FRP composites [12]. The use of epoxy adhesives for joining composite materials and debonding, and repairing civil structures is increasing [11,13]. Owing to the rapid cure time and high mechanical strength of epoxy, it is suitable for use in civil engineering applications [13], and composite materials with epoxy matrices are highly suitable for use in moist environments [14]. Wang et al. [15] found that stiffer carbon fibers lead to better mechanical properties for composites than neat resin. However, the fibers in composite materials age during service; therefore, degradation is likely [13,16]. Epoxy/CFRP composites have more versatile properties than other polymer matrices. However, during aging, they absorb water owing to the high number of polar hydroxyl groups within them [17].

The long-term service of carbon fiber-reinforced polymer (CFRP) in civil engineering structures requires more studies, as they are anticipated to be used for over 50 years. Furthermore, the intense usage of such composites would adversely affect their safety, economic efficiency, and potential applications. Therefore, the durability of CFRP composites must be studied more prior to application [7,10,18,19]. Environmental factors that include humidity, temperature, and the presence of aggressive media, such as water, acidic, and alkaline substances, play a significant role in the characterization of composite structures, particularly in civil engineering applications [5,11,20]. The unreacted resins in composites play an important role in the aging mechanism of polymer materials [21]. Additionally, degradation is a chemical issue occurring at the interface of the resin matrix, and may cause shrinkage, cracking, and molecular stiffening. Ramirez et al. [22] exposed a carbon fiber epoxy composite to different solutions, and found that moisture absorption occurs at the interface through cracks. Jiang et al. [19] reported that, when composite materials are exposed to moisture for a long time, their mechanical properties may decrease, thereby shortening their service life. Hence, environmental aging studies must focus on composite materials that are beneficial to civil engineering applications.

Accelerated aging studies of polymer composites under various environments assist in predicting the lifespan of the materials. Different durability analysis methods can be used depending on the exposure of the material, including hygrothermal aging, in which the degree of aging is determined via temperature, time, and ingress of water or any solution. Physical and chemical aging are the most common methods. Physical aging is reversible and arises in materials exposed to high temperatures for an extended period of time. Therefore, the molecular conformation results indicate increases in the thermal and mechanical properties. Chemical aging is irreversible and involves chain scission and degradation. This study focuses on the durability of neat epoxy and its CFRP composite under the conditions they may face in different engineering applications. We selected three types of corrosive environments, i.e., water, acidic, and alkaline, and different temperatures that are often encountered in civil applications.

Neat epoxy and CFRP composite durability studies have been conducted by other researchers; however, we also explored the matrix behavior during aging. Moreover, detailed aging analysis was conducted and the service life of CFRP composites was predicted under various aggressive conditions via the Arrhenius theory. The composites used for the aging studies were prepared by the vacuum-assisted resin infusion (VARI) molding process. The results of the hygrothermal aging of neat epoxy and composites in this study indicated that the aging pathways are very complex, and solution and temperature-dependent. The main aim of this study was to explore the aging behavior of materials under different conditions in order to achieve a better service life and economical design for engineering purposes. Thermal, mechanical, and morphological analyses were conducted for both aged and unaged neat resin and CFRP composites. Additionally, we predicted the long-term performance of the composite in different environments. The results of this study are mainly applicable to reinforcement, such as bonding and rehabilitation, in civil engineering.

## 2. Materials and Methods

### 2.1. Raw Materials

**Figure 1:**
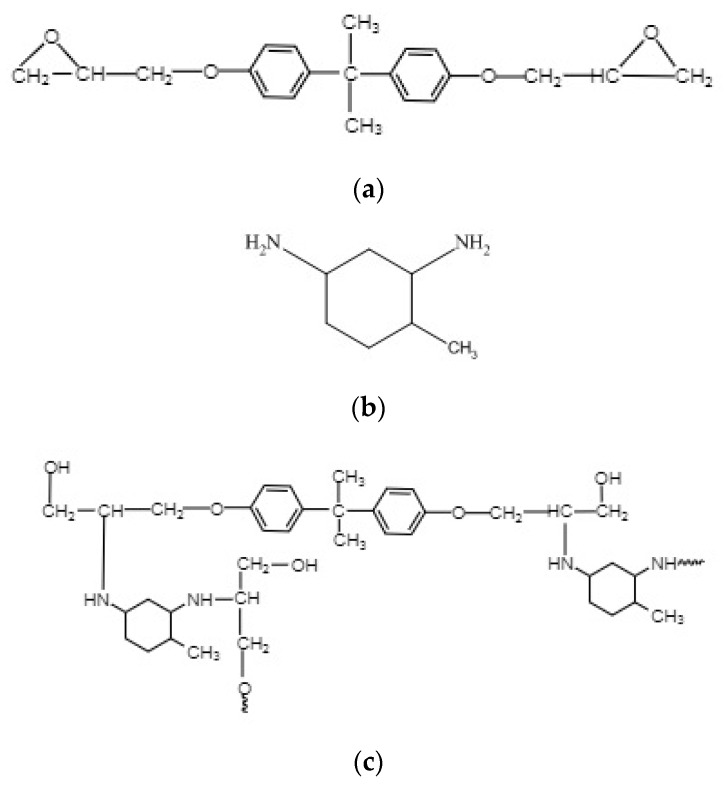
Chemical structures of the epoxy system used to prepare the neat epoxy and CFRP plates: (**a**) E51epoxy (**b**) HTDA and (**c**) cured epoxy resin.

In this study, we investigated the aging of an epoxy resin and carbon fiber-reinforced polymer (CFRP) composite under various environmental conditions. Bisphenol-A (E51) diglycidylether with a hardener of 4-methyl-1,3 cyclohexane diamine (HTDA) (Sinopec shanghai petrochemical., Shanghai, China) was used. Since as the E51 epoxy resin owing to its low water absorptivity, low viscosity, excellent mobility, and high mechanical strength. HTDA is also referred to as methyl cyclohexanediamine, which is an alicyclic amine-curing agent with a wide curing temperature range for epoxy resin with very low viscosity implemented in composite materials, coatings, and adhesives. The CFRP composite plates were fabricated from 300-g/m^2^ unidirectional carbon fabrics (Sinopec Shanghai petrochemical., Shanghai, China). Figure 1 presents the chemical structures of the epoxy system used to prepare the neat epoxy and CFRP plates.

### 2.2. Methods

#### 2.2.1. Preparation of Epoxy Resin Specimens

The resin specimens were prepared by thoroughly mixing the DGEBA (Diglycidylether Bisphenol-A, E51) epoxy and hardener. This mixture was then placed in a vacuum chamber to remove any air bubbles. The resin mixture was then poured into a mold for curing at room temperature (25 ± 1 °C) for 24 h and then post-cured for 2 h at 110 °C in a hot oven.

#### 2.2.2. Preparation of CFRP Plates

**Figure 2:**
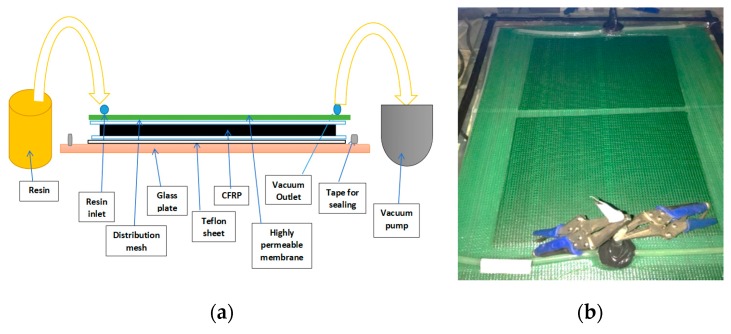
Illustration of vacuum-assisted resin infusion (VARI) molding process (**a**) Schematic and; (**b**) Photographic images.

The CFRP composite plates were prepared following the VARI molding process. The unidirectional carbon fabrics were stacked on a glass mold, and the epoxy resin with hardener was infused into the carbon fibers under a vacuum. The composite plates were then allowed to cure at room temperature (25 ± 1 °C) for 24 h and then post-cured in an oven for 2 h at 110 °C. Figure 2 presents schematic and photographic images of the VARI molding process.

### 2.3. Immersion Conditions

The neat epoxy and CFRP composite plates were tested for time intervals of 20, 40 and 80 days at submersion temperatures of 20, 40 and 60 °C. The details of the immersion conditions for the epoxy and CFRP composites are listed in Table 1. Distilled water was commercially purchased, while the alkaline solution was produced by dissolving sodium hydroxide (NaOH) into distilled water at a concentration of 10% by weight.

The acidic solution was produced by dissolving hydrochloric acid (HCl) in distilled water at a concentration of 5% by weight. The pH of alkaline and acidic solutions is about 13.0 and 1.0 respectively.

### 2.4. Characterization

#### 2.4.1. Dynamic Mechanical Analysis

The thermal properties of entire resin and composite materials were analyzed using dynamic mechanical analysis (DMA) with a Q800 system (TA Instruments Co Ltd., New Castle, DE, USA) in the dual cantilever beam mode. The temperature was increased from 20 °C to 250 °C at a ramp rate of 5 °C/min. The DMA measures the storage and loss moduli as functions of temperature. The glass transition temperature of the materials was determined from the tan delta peak.

#### 2.4.2. Tensile Test

The tensile properties of the epoxy resin and CFRP plates were tested using a universal mechanical testing machine (Jinan Shijin Group Co., Ltd., Jinan, China). Dog bone-shaped epoxy resin samples were used in the test. The mechanical properties of the epoxy resins were investigated according to ASTM D 638D at a loading rate of 1 mm/min. The mechanical properties of the CFRP plates were tested based on ASTM D 3039/D 3039M (Standard Test Methods for Tensile Properties of Polymer Matrix Composite Materials). The CFRP composite plates cut using a cutting machine and tested at a loading rate of 5 mm/min per ASTM D 3039. Five samples of both the neat epoxy and CFRP composite plates subjected to each condition were tested, and the average results are reported.

#### 2.4.3. Scanning Electron Microscopy

The morphologies of the aged and unaged neat epoxy and CFRP composites samples were analyzed. A Vega3 scanning electron microscope (Tescan, Brno, Czech Republic) was used to examine the morphology of the fracture surface of the materials. The samples were prepared by mounting a section from the tensile-fractured samples on an aluminum plate and coated with a layer of gold as a conducting material using an E5200 auto sputter coater (Cambridge, UK) before testing.

## 3. Results and Discussion

### 3.1. Dynamic Mechanical Analysis

**Figure 3:**
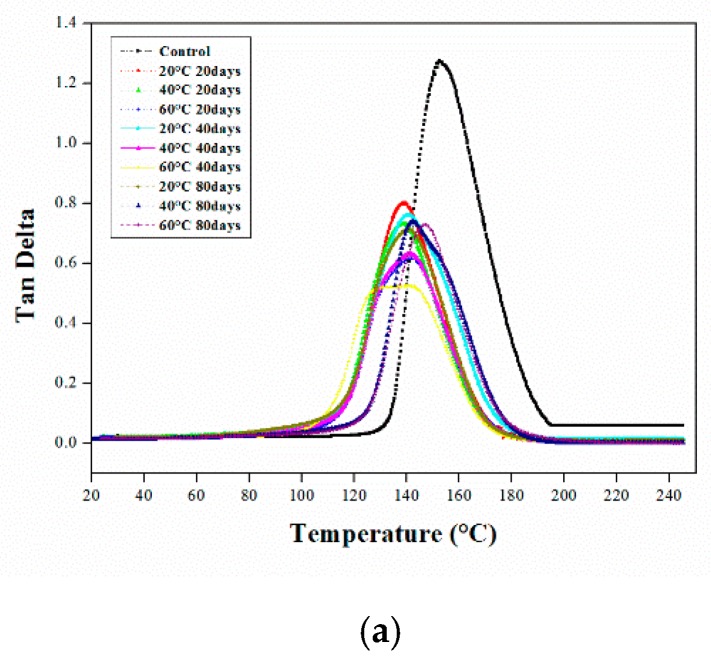

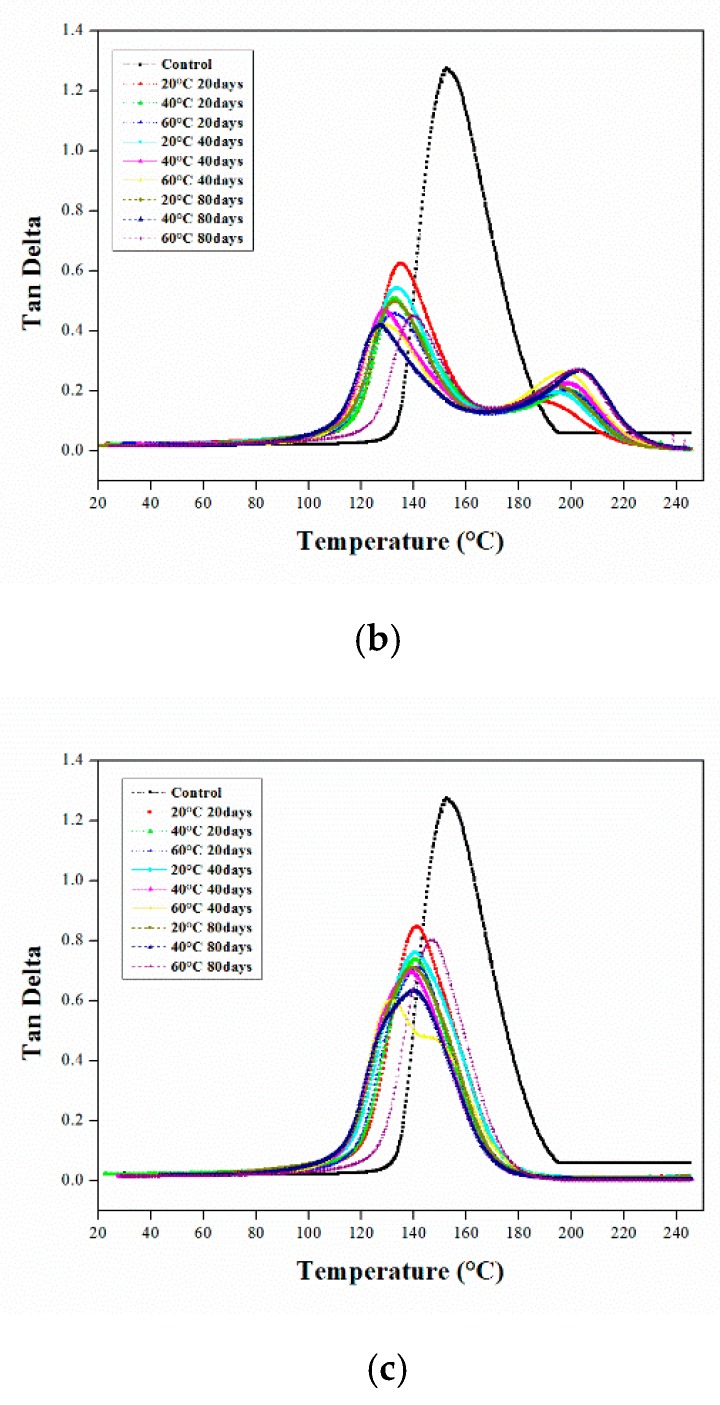
DMA tan delta curves of neat epoxy resins in different immersion temperatures (▫; control, ◦; at 20 °C, Δ; at 40 °C, +; at 60 °C) for (..; 20 days, -; 40 days, ---;80 days) in various solutions (**a**) Water; (**b**) HCl; and (**c**) NaOH solution.

**Figure 4:**
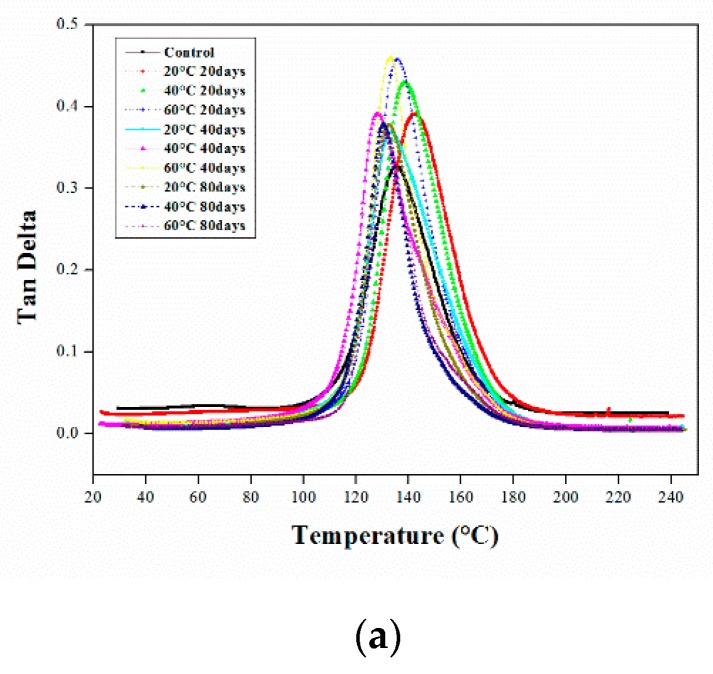

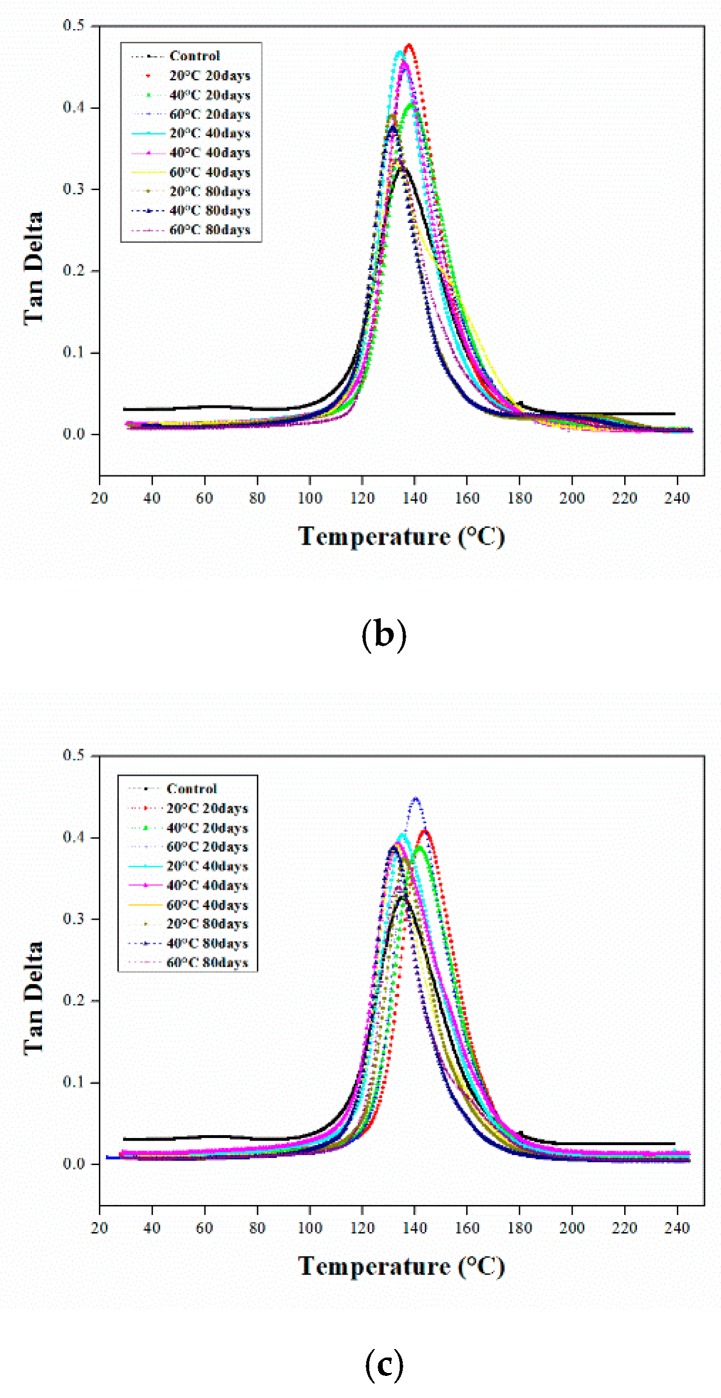
DMA tan delta curves of CFRP composites in different immersion temperatures (▫; control, ◦; at 20 °C, Δ; at 40 °C, +; at 60 °C) for (..; 20 days, -; 40 days, ---;80 days) in various solutions (**a**) Water, (**b**) HCl, and (**c**) NaOH solution.

The aged and unaged epoxy resin and CFRP composites were thermally analyzed by conducting a DMA test, and the results indicate that the neat resin and CFRP composite behaved differently under each aging condition. The glass transition temperature of the neat resin decreased greatly (Figure 3), while that of the composite materials only slightly decreased (Figure 4). The decrease in the glass transition temperature of a polymer material mainly depends on the degree of crosslinking; a high degree of crosslinking would decrease the chain mobility, and a high glass transition temperature indicates that a material is thoroughly cured. The glass transition temperatures (*T*_g_) of the epoxy resin and CFRP composites under different immersion conditions were evaluated using the relaxation peaks of the tan delta vs. temperature curves, which are summarized in Table 2.

#### 3.1.1. DMA of the Aged Epoxy Resins

As indicated by the DMA results, the intensity of the tan delta peaks of the aged samples decreased due to aging. The reduction of the tan delta curves in Figure 3 was greater under elevated temperatures than lower temperatures. Additionally, when the samples were aged for 80 days, the heights of the tan delta peaks were greatly reduced under soaking temperatures. After the DMA test, double peaks with a significant decrease in the glass transition temperature were observed in the tan delta vs. temperature plot for the samples immersed in the HCl solution. This might be due to the participation of the protons in the HCl in chemical reactions between water molecules via hydrogen bonding and within the hydrophilic groups of the epoxy, which resulted in the generation of a less-plasticized region dispersed across the surface of the polymer. According to previous studies, the irreversible degradation of aged materials is due to moisture or water uptake, which occurs due to plasticization [23] and results in a decrease in the glass transition temperature [24].

Figure 3a presents the tan delta curves of the neat epoxy against temperature upon immersion in water under various aging conditions. After 20 days, the glass transition temperature of the samples aged at 20 and 40 °C decreased by approximately 9%. However, at 60 °C, the glass transition temperature only decreased by 7%. This could be due to the post-curing reactions or the plasticization at elevated temperatures. The glass transition temperature of the samples aged for 40 days at 20 °C and 40 °C decreased by almost 7%, and that of the samples aged at 60 °C decreased by approximately 10%. At elevated temperatures, the great reduction in *T*_g_ was followed by material or polymer degradation due to the negative effects of plasticization. After 80 days of aging at 20, 40 and 60 °C, the glass transition temperatures of the neat epoxy samples decreased by approximately 8%, 6.8%, and 3.4%, respectively. This indicates that, after the initial aging periods, the rate of the reduction in *T*_g_ decreased. This is because the *T*_g_ of the materials typically decreased in the initial stage, and the reduction rate was reduced thereafter as the materials slowly became saturated with water or moisture [15]. Furthermore, as the aging continued, the post-curing reactions occurred and, when water absorption increased, the impact of plasticization was negligible [5].

The tan delta curves of the samples immersed in HCl are plotted against temperature under various aging conditions in Figure 3b. The most important finding is that secondary or double peaks occurred under all aging conditions. As mentioned above, the pseudo-crosslinking effect occurred. During the initial stage, the glass transition temperatures reduced by approximately 11% at 20 °C and 13% at 40 and 60 °C. As the aging time increased, the *T*_g_ decreased. After 40 days, the *T*_g_ decreased by 13% at 20 °C, and decreased by almost 15% at 40 and 60 °C. Similar to the results obtained for water, the glass transition temperature reduction rate was lowest after 80 days of immersion in HCl. The results indicated that the thermal properties of the samples were reduced by immersion in an acidic solution and at elevated soaking temperatures, indicating that both the immersion temperature and solution affected the materials and the occurrence of plasticization, molecular crosslink formation, and post-curing reactions [25,26].

The tan delta curves of the samples immersed in the NaOH solution are plotted against temperature under different aging conditions in Figure 3c. After immersion for 20 days, the glass transition temperatures decreased by 7% under all soaking temperatures. At 60 °C, the *T*_g_ decreased by 13% after 40 days of aging due to the effect of plasticization or the deterioration of materials at higher temperatures. After 80 days of soaking, the *T*_g_ of the samples slowly stabilized, similar to the water and HCl solutions. The *T*_g_ reduction rate under all aging conditions in the NaOH solution appeared to be close to those in water. The overall thermal analysis results indicate that the aged resin samples were mainly degraded in acidic environments.

#### 3.1.2. DMA of Aged CFRP Composites

The DMA of composite materials can indicate small-scale movements of the polymer-resin matrix over a wide range of temperatures. The DMA results indicate that the Tg of composites first increased, and was then similar to that of the control samples, remaining stable under all submersion conditions. Figure 4 plots the tan delta curves of the CFRP composites against temperature under different aging conditions. After 20 days of immersion in all solutions, the glass transition temperature increased, particularly in NaOH, where it increased by 6.5%. The increase in the glass transition was largely due to increases in the crosslinking density of the materials [24,27]. Additionally, the damping properties are vital when considering the tan delta peak of a material. After immersion in water for 20 days, the height of the tan delta curve in Figure 4a increased as the temperature increased. The *T*_g_ increased by approximately 4.7% and 2.28% at 20 °C and 40 °C, and at 60 °C, it was similar to that of the control samples. Upon immersion in HCl solution at 20 °C, 60 °C, and 40 °C, the height of the tan delta peak in Figure 4b increased. The glass transition temperature increased by approximately 2% at 40 °C. As shown in Figure 4c, the height of the tan delta peak for the samples immersed in the NaOH solution increased, similar to the HCl solution. The molecular-level penetration of NaOH molecules can restrict the segmental interactions, thereby improving *T*_g_ and decreasing the height of the tan delta peak [28]. *T*_g_ increased by approximately 6.43%, 4.88%, and 3.6% at 20, 40 and 60 °C, respectively, which was due to the effects of the post-curing and crosslinking reactions [5,29,30,31].

As shown in Figure 4a, when the samples were submerged in water for 40 days, the height of the tan delta curve of the composite specimens increased as the temperature increased, and the glass transition temperature decreased by approximately 2%. Figure 4b shows that the height of the tan delta peak of the samples immersed in the HCl solution at 60 °C was similar to that of the control samples. Moreover, the *T*_g_ of the samples immersed in HCl and NaOH at 20 °C and 40 °C was close to that of the control samples and decreased by approximately 2% at 60 °C, as shown in Figure 4c. Owing to the plasticization effect, *T*_g_ decreased at higher temperatures. However, the decrease in *T*_g_ observed here was only approximately 2%; therefore, the degradation of the resin matrix could be considered as negligible.

As shown in Figure 4, after 80 days of soaking in all solutions at all temperatures, the heights of the tan delta peaks increased and were almost similar, and the *T*_g_ values decreased by approximately 2%. This may have been due to the plasticization effect when the materials were submerged in solutions for an extended period of time and was negligible. Additionally, the heights of the tan delta peaks for the composites immersed at higher temperatures decreased, which may have been due to the slow curing rate at higher temperatures over time. Therefore, the improvement in the overall crosslink density decreased the segmental molecular chain mobility of the polymer matrix, resulting in low energy loss. Acidic media are also highly corrosive environments, and the composites subjected to the acidic solution were dehydrated due to the diffusion of the solution into the polymer matrix and the carbon fiber surface. This penetration is either activated by the presence of micro-cracks on the surface of the composite or manufacturing defects.

### 3.2. Tensile Properties

The effects of moisture on the tensile properties of the neat epoxy resin and its CFRP plates upon immersion in water, acidic, and alkaline solutions for 80 days were tested to determine the mechanical properties of the composites as they play a vital role in their use in engineering applications. The strength of composite materials affects their service life, and the tensile strength and modulus should be determined to improve the service life and the design of materials used in civil engineering. The degradation of the materials affected the mechanical properties of both the aged neat resin and composites. The influence of moisture and temperature on the tensile properties of the materials aged with a long immersion time may have been due to the deterioration process [32]. Zhang et al. [33] found that moisture may either increase or decrease the tensile strength of composite materials. In our study, the tensile strength of both the resin and composites decreased, while the modulus did not significantly change. Table 3 presents the reduction (%) in tensile strength, and Table 4 presents the reduction (%) in the tensile modulus of the epoxy resin and CFRP composites under various immersion conditions.

#### 3.2.1. Tensile Properties of Aged Epoxy Resins

**Figure 5:**
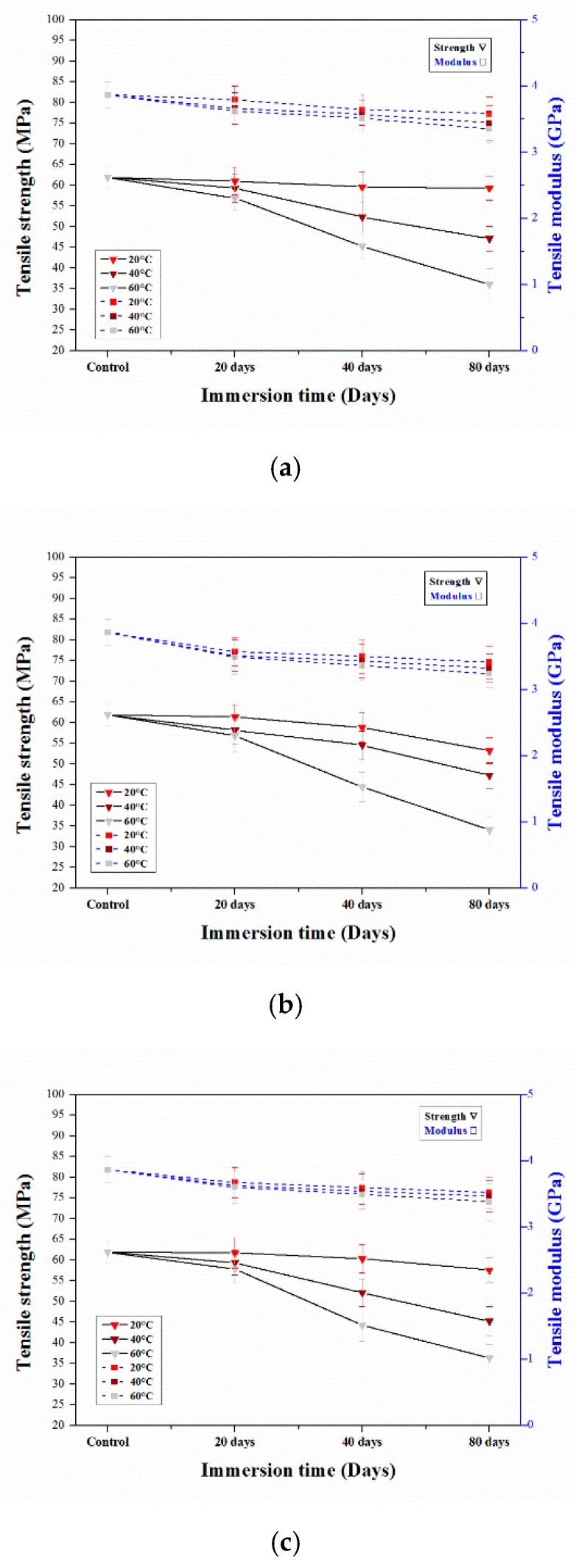
Tensile strength and modulus vs. immersion days of neat epoxy resin in different immersion temperatures (**a**) Water (**b**) HCl and (**c**) NaOH solution.

The tensile test results showed that the mechanical properties of the neat epoxy resin decreased during aging. Figure 5 shows the tensile strength and moduli of the aged and unaged epoxy resins under various conditions. The initial tensile strength of the epoxy resin was 61.9 MPa and, when the resin samples were immersed in water at 20 °C, the initial tensile strength decreased to 61 MPa, 59.57 MPa, and 59.3 MPa after 20, 40 and 80 days, respectively (Figure 5a). The reduction in the tensile strength was mainly due to the effect of plasticization. Additionally, decreases in the tensile strength occurred as water penetrated the resin in the deterioration process. Upon immersion at 40 °C, the tensile strength of the samples decreased to 59.32, 52.33 and 47.13 MPa after 20, 40 and 80 days, respectively. Moreover, at 60 °C, the tensile strength decreased to 56.89, 45.21 and 36 MPa after 20, 40 and 80, respectively.

The strength of the resin samples immersed in HCl at different temperatures exhibited a greater decrease, particularly for immersion at 60 °C. As shown in Figure 5b, after soaking in HCl at 60 °C for 20 days and 40 days, the tensile strength declined to 56.89 and 44.42 MPa, which were decreases of 8.1% and 28.3% from the initial value. Furthermore, higher degradation was observed after 80 days of immersion; the tensile strength decreased by 45% to 34.05 MPa. The strength of the resin samples soaked at 20 °C for 20, 40 and 80 days decreased to 61.34, 58.81 and 53.23 MPa, respectively. Furthermore, at 40 °C, the tensile strength decreased to 58.15, 54.56 and 47.23 MPa after 20, 40 and 80 days, respectively. The decreases in tensile strength were due to the deterioration of the material and the formation of cracks due to the acid [34] or moisture uptake.

Figure 5c shows that the tensile strength of the resin immersed in NaOH changed under all soaking conditions. The strength of the resin immersed at 20 and 40 °C decreased to 61.69 MPa and 59.35 MPa after 20 days of immersion, respectively. After immersion at 20 and 40 °C, for 40 days, the strength of the resin decreased to 60.29 and 52.12 MPa, respectively. Moreover, after aging for 80 days, the tensile strength decreased to approximately 57.57 and 45.21 MPa, respectively. After soaking at 60 °C for 20, 40 and 80 days, the strength decreased to 57.83, 44.25, and 36.33 MPa, respectively, which are decreases of 6.6%, 28.5%, and 41.3% from the initial value. Bin et al. [35] reported that, at higher immersion temperatures, the decrease in tensile strength was due to the plasticization effect. The tensile modulus values of the epoxy resin samples soaked in water for 80 days at 20, 40 and 60 °C decreased by approximately 7.34%, 10.97%, and 13.3%, while those of the samples soaked in the HCl solution decreased by 11.74%, 14.07%, and 16.14%, respectively. The tensile moduli of the samples immersed in the NaOH solution decreased by 8.9%, 10.45%, and 12.52% after 80 days of soaking at 20, 40 and 60 °C, respectively. Table 2 shows that the degradation of the tensile modulus of the neat epoxy was lower than that of the tensile strength.

#### 3.2.2. Tensile Properties of Aged CFRP Composites

**Figure 6:**
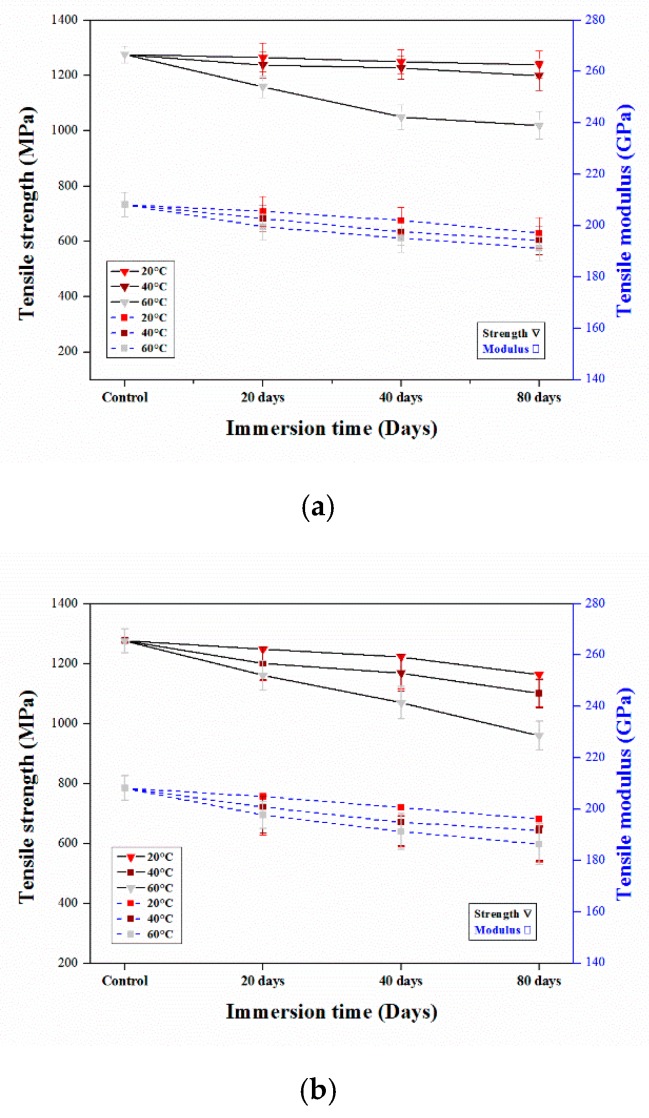

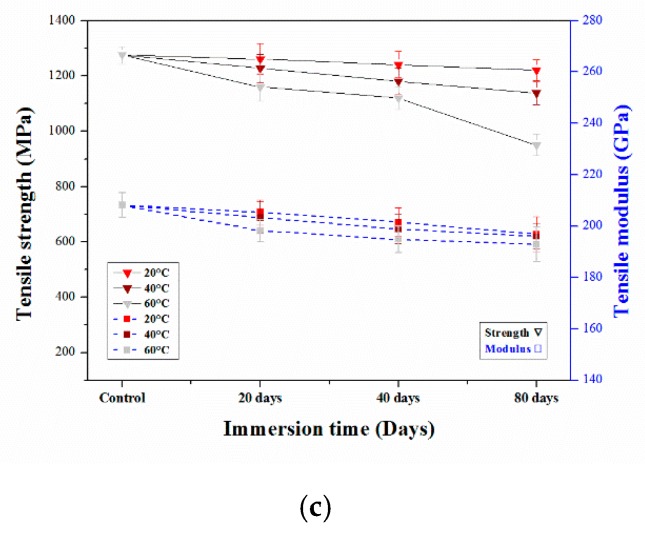
Tensile strength and modulus vs. immersion days of CFRP composite in different immersion temperatures (**a**) Water (**b**) HCl and (**c**) NaOH solution.

The results of this study indicated that the addition of carbon fibers had little impact on the moisture absorption or degradation of the composites. The epoxy matrix caused stress to the interface; subsequently, debonding and cracking occurred at the fiber-matrix interphase. The unaged and aged composites exposed to various environments were mechanically tested, and the results are presented in Figure 6.

**Figure 7:**
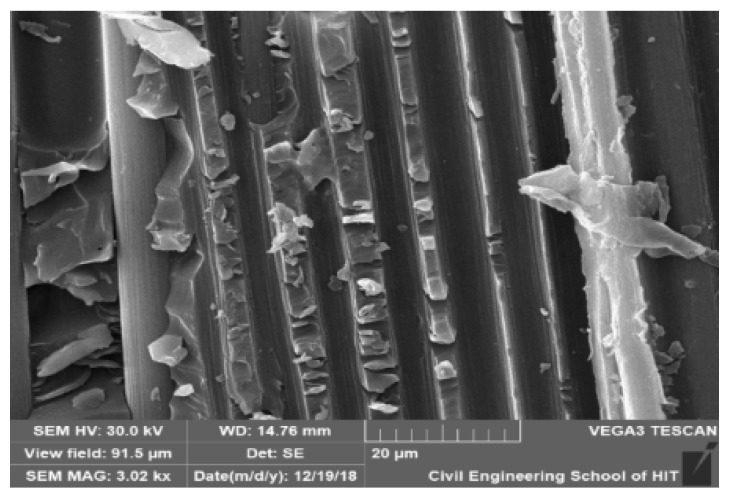
SEM image of the polymer matrix degradation of the composite in HCl solution.

During the initial stage (20 days) of aging, the tensile strength and modulus of the composites decreased slightly, and after 40 and 80 days, degradation mainly increased in the HCl solution, which could be attributed to the corrosion of the polymer matrix and the cracks that may have been formed by the residual thermal stress during fabrication, which allowed the HCl to reach the fibers [36]. The tensile tests indicate that, during hygrothermal aging, the immersion solution greatly affects the mechanical properties as all composites behaved differently in water, NaOH, and HCl. The degradation details of tensile strength and modulus of CFRP materials is shown in Table 3 and Table 4. From the table we can clearly understand that in HCl solution, especially at 60 °C the mechanical properties decreased. Sindhu et al. [28] found that the tensile strength and Young’s modulus of natural and glass fibers increased upon long-term immersion in a HCl solution, but decreased upon immersion in NaOH. According to Somjai et al. [26], immersion in an acidic solution at 60 °C and room temperature affected the surface of the material, and the mechanical properties were damaged by water and alkaline solutions. However, Amaro et al. [37] found that immersion in an alkaline solution resulted in a greater decrease in the mechanical properties of a material than immersion in an acidic solution. The mechanical properties can be influenced by the fracture mechanism [38]. The degradation of the polymer matrix of the composite samples in the HCl solution is presented in the SEM image in Figure 7, which shows the damage, cracks, and deterioration that occurred in the matrix.

The initial tensile strength of the composite was 1276.1 MPa. Upon immersion in water, HCl, and NaOH solutions for 80 days at 60 °C, the tensile strength decreased by approximately 20.06%, 24.77%, and 23.98%, respectively. The exposure of the composite materials to moisture caused the decrease in the mechanical properties [29]. The material toughness indicated that the changed in the mechanical properties during treatment was caused by volatile matter, such as water molecules, and that it reduced the plasticity or oxidation of the molecules in the materials [39,40]. The changes were attributed to the cross-linking effect upon exposure to higher temperatures. The tensile modulus of the composites also decreased upon immersion at 60 °C by approximately 10%, 8%, and 7% in HCl, water, and NaOH solutions, respectively. As the aging temperature and duration increased, mechanical degradation increased [23,41].

From the thermal and mechanical analysis, we perceived that the rate of degradation is accelerated with increase in temperature. In addition, the main correlation observed during the DMA and tensile test is that the decrease in properties is observed higher in acidic environments. From the thermal analysis of CFRP samples it is observed that during initial stages, the *T*_g_ increased in all immersion conditions, this can be attributed to the increase in crosslink density of the material. On co-relating the cross-linking effect to the tensile properties of CFRP samples the overall the rate of degradation is also observed at a lower rate during the initial stages. The degradation in tensile strength and DMA may ascribed due to the plasticization effect of the materials.

### 3.3. Service Life Prediction of the CFRP Composite in the Three Solutions

The service life of composite materials must be understood when considering civil engineering applications. Our experimental results indicated the degradation of the aged materials. Therefore, we predicted the service life of the composites in water, acidic, and alkaline solutions using the Arrhenius theory.

#### 3.3.1. Arrhenius Relationship

According to the Arrhenius relationship, the rate of degradation can be expressed [42,43,44] as Equation (1):

(1) $$k=A\;\exp(-E_{a}/RT)$$

where k is the degradation rate, i.e., 1/time, A is the material constant, $E_{a}$ is the activation energy, R is the universal gas constant, and T is the temperature (Kelvin). The Arrhenius relationship assumes that, during aging, the sole leading degradation mechanism does not change over time and with changes in temperature. However, the rate of degradation increased as the temperature increased.

Equation (1) can be transformed into Equations (2) and (3):

(2) $$\frac{1}{k}\;=\;\frac{1}{A}\;\exp(-E_{a}/RT)$$

(3) $$\ln\left(\frac{1}{k}\right)\;=\;\;\frac{E_{a}}{RT}\frac{1}{T}\;-\;\ln\;A$$

Equation (2) represents the rate of degradation, where k is the inverse of the time required for a material property to reach a given value. Equation (3) is the logarithm of the time required for a material property to reach a given value, and is a linear function of 1/T with a slope value of $E_{a}/RT$.

#### 3.3.2. Degradation Prediction Procedure

In this study, the tensile properties of the accelerated aged CFRP composites subjected to three different immersion media (water, acid, and alkali) at three different temperature ranges were predicted using the degradation model given in Equation (4), which defines the relationship between the tensile strength retention of the CFRP composite and the exposure time in the accelerated aging study:

(4) $$Y=100\exp\left(\frac{-t}{\tau }\right)$$

where *Y* is the tensile strength retention (%), t is the exposure/aging time, and τ is the fitted parameter.

**Figure 8:**
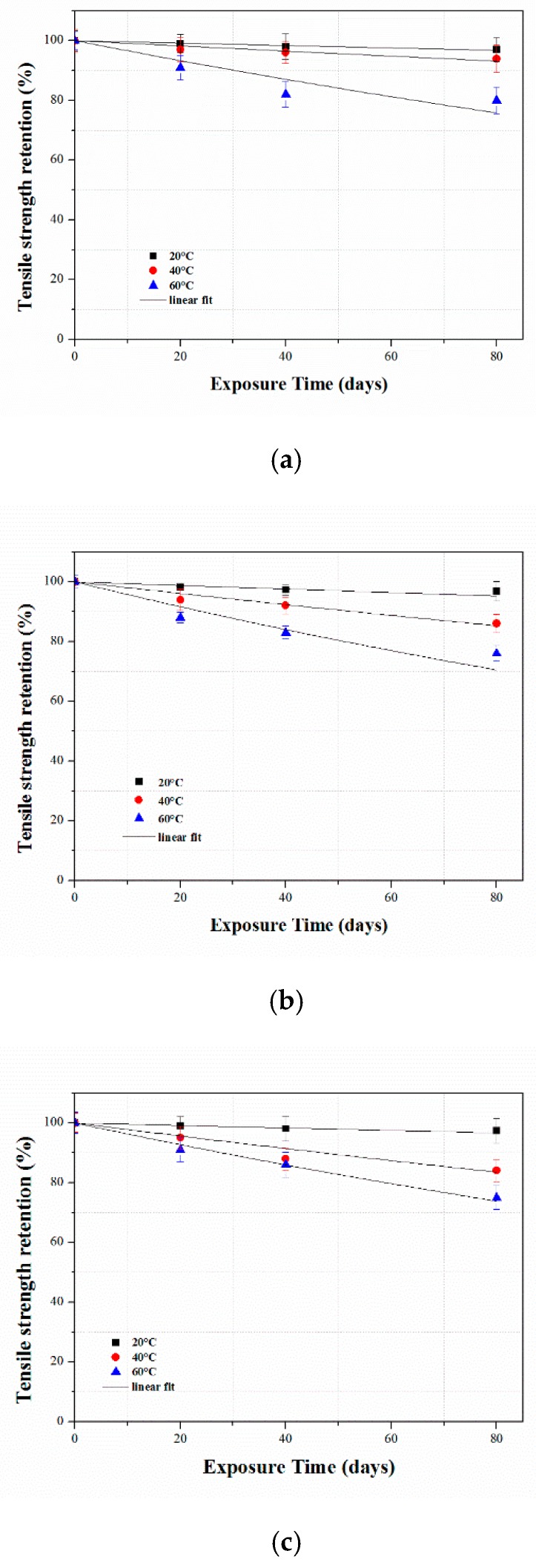
Predictions of CFRP composite at given temperatures (20 °C, 40 °C and 60 °C) in different immersion solutions (**a**) Water, (**b**) HCl and (**c**) NaOH solution.

The experimental values were applied to Equation (4) and the results are plotted in Figure 8. The values of τ and the correlation coefficient $R^{2}$ for the three different test conditions are given in Table 5.

**Figure 9:**
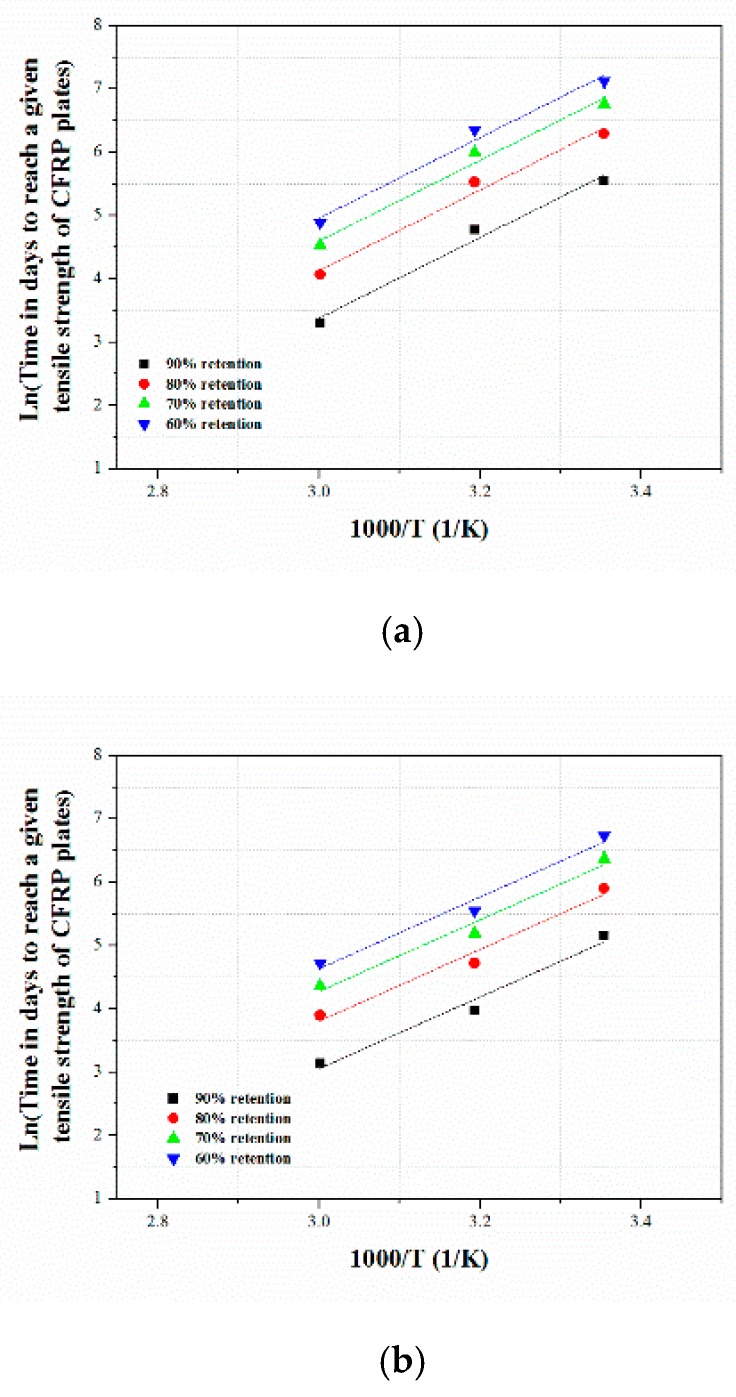

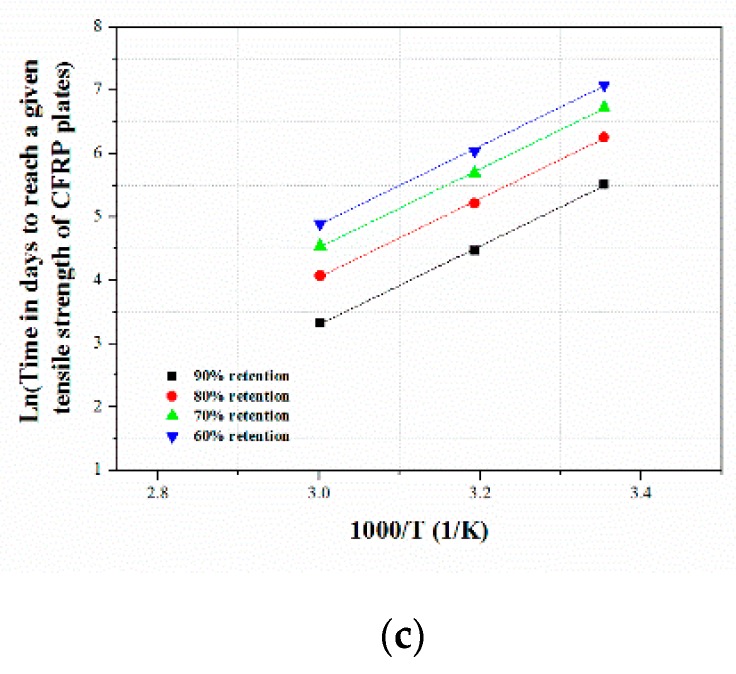
Arrhenius plot of tensile strength retention of CFRP composite in different solutions (**a**) Water, (**b**) HCl and (**c**) NaOH solution.

In the second step, by applying the regression coefficient τ, the time (*t*) required for the tensile strength retention to reach 60%, 70%, 80%, and 90% at temperatures of 20, 40 and 60 °C was calculated, and the obtained values were then fitted to Equation (3). The relationships between the results and ln(1/k) =lnt and 1/T are shown in Figure 9. By plotting the results of Equation (3), we acquired values that were parallel straight lines. The values of $E_{a}/RT$ are the slopes of the straight lines, and the correlation coefficients are given in Table 6.

In the third step, the time shift factor (TSF) to reach the same tensile strength values (represented by c) at temperatures $T_{0\;}$ and $T_{1\;}$ was determined from the previous Arrhenius plots.

The time-shift factor (TSF) can be calculated as:

(5) $$TSF\;=\;\frac{t_{0}}{t_{1}}\;=\;\frac{c/k_{0}}{c/k_{1}}\;=\;\frac{A\exp\left(-E_{a}/RT_{1}\right)}{A\exp\left(-E_{a}/RT_{0}\right)}$$

(6) $$TSF\;=\;\exp\left[\frac{E_{a}}{R}\left(\frac{1}{T_{0}}\;-\;\frac{1}{T_{1}}\right)\right]$$

To predict the long-term behavior of the CFRP composites in this study, we have selected five reference temperatures from cities in Canada. In Equation (6), the reference temperature $T_{0\;}$ is the annual mean temperature of the selected cities. The TSF of the composites at different temperatures was calculated, and the results are presented in Table 7.

**Figure 10:**
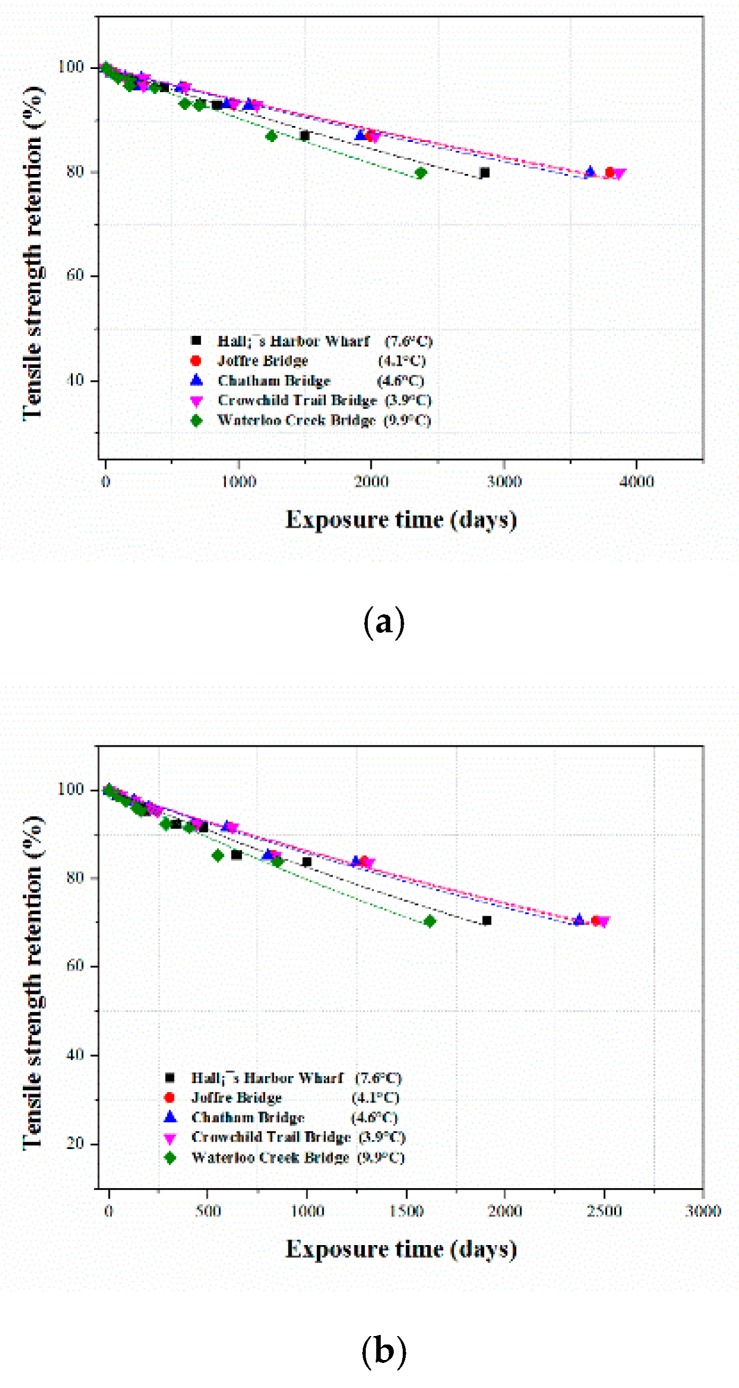

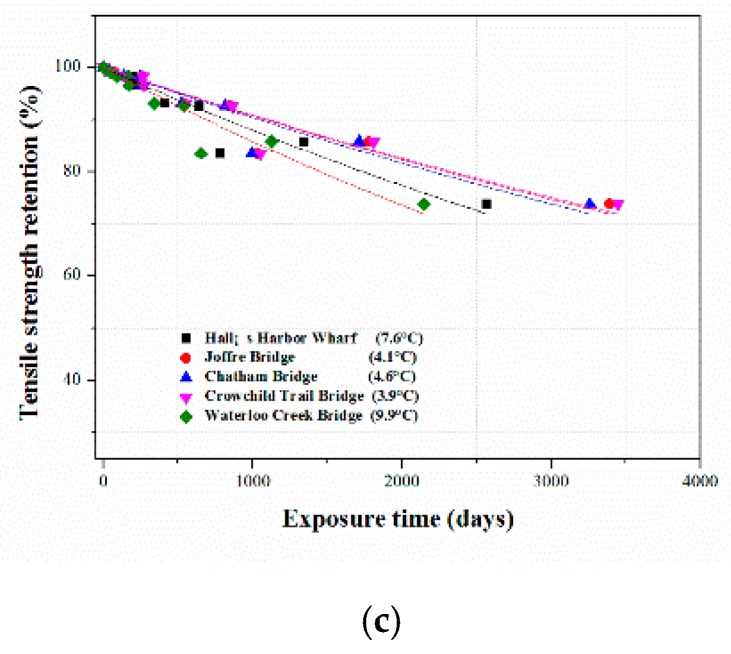
Master curves of CFRP composite exposed to different annual temperatures (**a**) Water, (**b**) HCl and (**c**) NaOH solution.

The master curves of the relationship of the tensile strength retention of the CFRP composites with exposure time in the five selected cities with five different annual temperatures obtained under the different TSF values at 20, 40 and 60 °C were determined, and, based on the annual service temperatures, Figure 9 was transformed into Figure 10. The results of the fitted master curves for all immersion media are summarized in Table 8.

#### 3.3.3. Long-Term Tensile Strength Retention Prediction

The time in years required to reach a selected tensile strength retention value, i.e., 70%, for the CFRP composites immersed in water, acidic, and alkaline solutions under the five service temperatures selected from Canada was predicted and is presented in Table 9. Under the five selected annual service temperatures, it is expected that it will take 9.7–15.8, 4.3–6.7, and 8.3–10.2 years for the tensile strength retention of the CFRP composites to reach 70% in water, acidic, and alkaline solutions. The analysis indicated that the CFRP composites were more durable in water than acidic and alkaline solutions. Furthermore, the CFRP composites were less durable in acidic solutions and are more susceptible to degradation in acid.

Table 10 lists the predicted tensile strength retention values of the CFRP composites in the three media studied here under the selected five annual service temperatures. The tensile strength retention values were calculated using Equation (4) by substituting the values of τ with those given in Table 8, corresponding to the five average annual service temperatures. The prediction results indicated that the tensile strength retention values varied from 63.6–48% for the samples immersed in water for 20 years. The durability of the samples in the alkaline medium was better than that in acid and lower than that in water, and ranged from 49.8–32.7%. The CFRP composites exhibited low durability in the acidic solution, with the tensile strength retention after 15 years ranging from 44.9–29.1%.

Following the experimental study and theoretical analysis based on the Arrhenius relationship, we can conclude that the CFRP composites tested in this study were more susceptible to acid degradation than degradation in alkaline solutions and water. The fitted curves of the prediction of the long-term tensile strength using the Arrhenius relationship (Table 8) had correlation coefficients of 0.97, 0.96, and 0.91 for water, acidic, and alkaline media, indicating the consistency of this analysis.

### 3.4. Morphological Analysis

**Figure 11:**
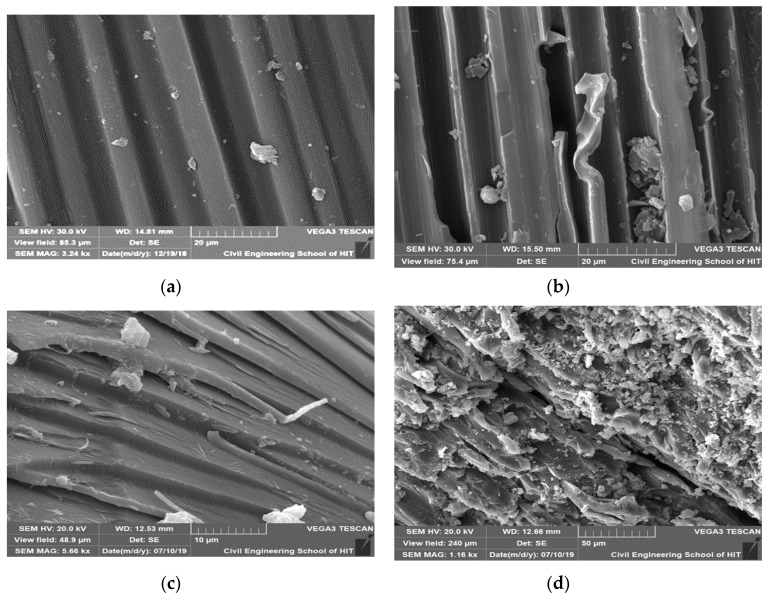
SEM images of the specimens (**a**) unaged CFRP composite, tensile fractured surface of (**b**) aged CFRP composites in HCl solution at 60 °C for 80 days, (**c**) unaged epoxy resin and (**d**) aged epoxy resin in HCl solution at 60 °C for 80 days.

The morphology of the neat epoxy and composite tensile test samples was examined based on the fracture mode by scanning electron microscopy. The main degradation of the aged resin and composites upon immersion in the HCl solution is shown in Figure 11. The SEM images show the breakage of fibers and debonding of the interphase matrix and voids. The cracking [34], debonding at the interphase matrix region, and shrinkage can enhance degradation for a long aging time at elevated temperatures [45,46]. Following thermal and mechanical analysis, we observed that the properties reduced and the materials degraded. The morphological analysis results can be used to understand the main degradation phases for the neat resin and CFRP composites immersed in the HCl solution. The role that the epoxy matrix plays in epoxy/CFRP composites during hygrothermal aging can be detected from the SEM images. The carbon fibers neither absorbed or swelled in the solutions, while the epoxy matrix swelled due to the stress at the interphase, resulting in cracking and debonding [47,48]. The cracks indicated the increased degradation kinetics in high-stress situations [32]. Acid caused more damage at 60 °C than that at room temperature [26]. The deterioration of the composite material was not solely due to plasticization, although hydrolysis and the debonding of the fiber-matrix interphase resulted in the densification of microcracks at higher submergence temperatures. This indicates that the acid solution reached the surface of the fibers and attacked their structure. Figure 11c,d show the degradation of resin and the moisture ingress for the aged and unaged neat resin. The SEM images of the composite samples indicated that the resin matrix area was more affected by degradation than the fibers.

## 4. Conclusions

In this study, we explored the aging of neat epoxy and its CFRP composite in water, acidic, and alkaline solutions at different temperatures. The long-term service life of the CFRP composites was also predicted from the tensile strength retention values under different environmental conditions by the Arrhenius theory.

The DMA results showed that the glass transition temperature initially increased and the height of tan delta peak decreased for the CFRP composites; however, the glass transition temperature of the neat epoxy samples decreased, and a double peak was observed for the samples immersed in the HCl solution. The tensile tests revealed that degradation adversely affected the tensile strength, although the tensile modulus values did not significantly decrease throughout the aging study. According to the thermal and mechanical analysis, degradation occurred in a higher rate as the exposure temperature is higher. In general, from the thermal and mechanical analysis it was observed the rate of degradation is accelerated at elevated temperature and is obvious in acidic conditions.

The degradation of the composite materials can be attributed to the deterioration of the resin matrix and debonding at the fiber-resin interface. This damage was visible in the morphological analysis. The potential relation of temperature and time-dependency of the degradation rate of the composite in different solutions were predicted using the Arrhenius equation, and the service life of the CFRP composites in the HCl solutions was shorter. By correlating the experimental data and prediction results, it can be concluded that the CFRP composite materials may need to be developed further to withstand acidic media and ensure a longer service life.

## Figures and Tables

**Table 1:** Shows the details of immersion conditions applied for the epoxy and CFRP composite plates in the aging study.

Solutions	Temperatures (°C)	Duration (Days)
Water	20/40/60	20/40/80
Acid (HCl)	20/40/60	20/40/80
Alkaline (NaOH)	20/40/60	20/40/80

**Table 2:** Glass transition temperature (*T*_g_ ± 1 °C) of epoxy resin and CFRP composites in different immersion conditions.

Samples	Immersion Time (Days)	Water			HCl			NaOH		
		20 °C	40 °C	60 °C	20 °C	40 °C	60 °C	20 °C	40 °C	60 °C
Epoxy resin C ^1^: 152.8	20	139.0	138	141.7	135.5	132.52	133	140.8	141.0	141.3
	40	141.0	141.3	136.2	133.6	129.3	129.0	141.0	139.1	131.8
	80	140.6	142.4	147.6	132.6	127.0	139.9	139.5	141.0	147.0
CFRP C ^1^: 135.8	20	142.3	139.0	135.8	137.9	139.0	136.7	144.6	142.5	140.7
	40	133.1	128.1	132.7	135.0	136.2	132.6	135.7	135.1	132.7
	80	133.0	130.5	132.1	131.6	132.0	133.1	132.2	133.0	133.4

^1^ C is the control or unaged sample.

**Table 3:** Tensile strength reduction (%) of the epoxy and CFRP composites at different immersion conditions.

Samples	Immersion Time (Days)	Water			HCl			NaOH		
		20 °C	40 °C	60 °C	20 °C	40 °C	60 °C	20 °C	40 °C	60 °C
Epoxy resin	20	1.5	4.2	8.09	1	6.1	8.1	0.3	4.1	6.6
	40	3.8	15.5	27	5	11.9	28.3	2.6	15.8	28.5
	80	4.2	23.9	41.8	14	23.7	45	7	27	41.3
CFRP	20	0.9	3.0	9.1	2.2	6.0	9.0	1.2	3.8	10.0
	40	2.0	4.0	17.7	4.2	8.4	16.2	2.8	7.5	12.2
	80	2.8	6.0	20.0	9.0	14.0	25.0	4.3	10.8	24.0

**Table 4:** Tensile modulus reduction (%) of the epoxy and CFRP composites at different immersion conditions.

Samples	Immersion Time (Days)	Water			HCl			NaOH		
		20 °C	40 °C	60 °C	20 °C	40 °C	60 °C	20 °C	40 °C	60 °C
Epoxy resin	20	1.9	5.3	6.3	7.6	9.2	10.0	5.0	6.3	6.8
	40	6.0	7.6	9.2	9.4	11.2	13.0	7.1	8.4	10.0
	80	7.3	11.0	13.3	11.7	14.1	16.1	8.9	10.5	12.5
CFRP	20	1.3	3.0	4.1	1.6	3.5	5.0	1.4	2.3	5.0
	40	2.9	5.1	6.3	3.6	6.4	8.1	3.2	4.5	6.4
	80	5.3	7.0	8.1	6.0	7.9	10.5	5.4	6.0	7.4

**Table 5:** Coefficients of regression equations in Equation (4).

Immersion Solutions	Temperatures (°C)	*τ*	*R* ^2^
Water	20	2425	0.95
	40	1127	0.87
	60	288	0.86
HCl	20	2341	0.87
	40	445	0.96
	60	263	0.97
NaOH	20	1641	0.91
	40	503	0.92
	60	240	0.91

**Table 6:** Coefficient of regression equation in Equation (5) for Arrhenius plots.

Immersion Solutions	Tensile Strength Retention (%)	*E*_a_/*R*	*R* ^2^
Water	60	6379	0.96
	70	6379	0.96
	80	6379	0.96
	90	6379	0.96
HCl	60	5660	0.95
	70	5660	0.95
	80	5660	0.95
	90	5660	0.95
NaOH	60	6197	0.99
	70	6197	0.99
	80	6197	0.99
	90	6197	0.99

**Table 7:** Time-shift factor of CFRP composites at different annual temperature chosen.

Immersion Solutions	Immersion TEMPERATURES (°C)	Time Shift Factor (TSF)				
		Hall’s Harbor Wharf 7.6 °C	Joffre Bridge (4.1 °C)	Chatham Bridge (4.6 °C)	Crowchild Trail Bridge (3.9 °C)	Waterloo Creek Bridge (9.9 °C)
Water	20	2.61	3.48	3.34	3.54	2.17
	40	10.50	13.99	13.42	14.22	8.73
	60	35.67	47.52	45.59	48.32	29.66
HCl	20	2.35	3.03	2.92	3.07	1.99
	40	8.05	10.39	10.01	10.54	6.84
	60	23.83	30.74	29.63	31.19	20.23
NaOH	20	2.54	3.36	3.23	3.41	2.12
	40	9.80	12.94	12.43	13.15	8.19
	60	32.10	42.41	40.74	43.10	26.84

**Table 8:** Coefficient of regression equations for the master curves of CFRP composites under various immersion conditions.

Conditions	Reference Temperature Cites	Average Annual Temperature (°C)	*τ*	*R* ^2^
Water	Hall’s Harbor Wharf	7.6	11,927	0.97
	Joffre Bridge	4.1	15,890	0.97
	Chatham Bridge	4.6	15,246	0.97
	Crowchild Trail Bridge	3.9	16,156	0.97
	Waterloo Creek Bridge	9.9	9916	0.97
HCl	Hall’s Harbor Wharf	7.6	5218	0.96
	Joffre Bridge	4.1	6731	0.96
	Chatham Bridge	4.6	6487	0.96
	Crowchild Trail Bridge	3.9	6831	0.96
	Waterloo Creek Bridge	9.9	4430	0.96
NaOH	Hall’s Harbor Wharf	7.6	7807	0.91
	Joffre Bridge	4.1	10,313	0.91
	Chatham Bridge	4.6	9907	0.91
	Crowchild Trail Bridge	3.9	10,483	0.91
	Waterloo Creek Bridge	9.9	6524	0.91

**Table 9:** Long- term prediction results of CFRP composites at different mean annual temperatures.

Reference Temperature Cites	Average Annual Temperature (°C)	Time in Years to Reach 70% Tensile Strength Retention of CFRP Composites		
		CFRP Composites in Water	CFRP Composites in Acid Medium	CFRP Composites in Acid Medium
Hall’s Harbor Wharf	7.6	11.7	5.1	7.6
Joffre Bridge	4.1	15.5	6.6	10.1
Chatham Bridge	4.6	14.9	6.3	9.7
Crowchild Trail Bridge	3.9	15.8	6.7	10.2
Waterloo Creek Bridge	9.9	9.7	4.3	8.3

**Table 10:** Prediction of tensile strength retention at different annual temperatures for 25 years.

Immersion Solutions	Time (Years)	Time in Years to Reach 70% Tensile Strength Retention of CFRP Composites				
		Hall’s Harbor Wharf	Joffre Bridge	Chatham Bridge	Crowchild Trail Bridge	Waterloo Creek Bridge
Water	5	86	89.1	88.7	89.3	83.2
	10	73.6	79.5	78.7	79.8	69.2
	15	63.2	70.9	69.8	71.3	57.6
	20	54.2	63.2	62	63.6	48
HCl	5	70.5	76.3	75.5	76.6	66.2
	10	49.7	58.1	57	58.6	44
	15	35	44.3	43	44.9	29.1
NaOH	5	79.2	83.8	83	84	75.6
	10	62.7	70.2	69.2	70.6	57.2
	15	49.6	58.8	57.5	59.3	43.2
	20	39.3	49.3	47.9	49.8	32.7

## References

[1] Guzmán E., Cugnoni J., Gmür T. (2014). Multi-factorial models of a carbon fibre/epoxy composite subjected to accelerated environmental ageing. Compos. Struct..

[2] Soutis C. (2014). Introduction: Engineering Requirements for Aerospace Composite Materials. Polymer Composites in the Aerospace Industry.

[3] Brunner A.J. (2014). Fracture Mechanics Characterization of Polymer Composites for Aerospace Applications. Polymer Composites in the Aerospace Industry.

[4] Awad Z.K., Aravinthan T., Zhuge Y., Gonzalez F. (2012). A review of optimization techniques used in the design of fibre composite structures for civil engineering applications. Mater. Des..

[5] Xian G., Karbhari V.M. (2007). DMTA Based Investigation of Hygrothermal Ageing of an Epoxy System Used in Rehabilitation. J. Appl. Polym. Sci..

[6] Xiao B., Li H., Xian G. Hygrothermal Ageing of Basalt Fiber Reinforced Epoxy Composites. Proceedings of the CICE 2010—The 5th International Conference on FRP Composites in Civil Engineering.

[7] Xian G., Li H., Su X. (2013). Effects of immersion and sustained bending on water absorption and thermomechanical properties of ultraviolet cured glass fiber-reinforced acylate polymer composites. J. Compos. Mater..

[8] Grammatikos S.A., Evernden M., Mitchels J., Zafari B., Mottram J.T., Papanicolaou G.C. (2016). On the response to hygrothermal aging of pultruded FRPs used in the civil engineering sector. Mater. Des..

[9] Dharsini S.P., Bhuvaneshwari B., Palani G.S., Ganesh G.M., Iyer N.R. (2014). FEA Studies on the Interfacial Behavior of Epoxy-CFRP Composites. J. Civ. Eng. Res..

[10] Karbhari V.M., Abanilla M.A. (2007). Design factors, reliability, and durability prediction of wet layup carbon/epoxy used in external strengthening. Compos. Part B.

[11] Gude M.R., Prolongo S.G., Ureña A. (2013). Hygrothermal ageing of adhesive joints with nanoreinforced adhesives and different surface treatments of carbon fibre/epoxy substrates. Int. J. Adhes. Adhes..

[12] Jin F., Lee S., Park S. (2013). Polymer matrices for carbon fiber-reinforced polymer composites. Carbon Lett..

[13] Stewart A., Douglas E.P. (2012). Accelerated Testing of Epoxy-FRP Composites for Civil Infrastructure Applications : Property Changes and Mechanisms of Degradation. Polym. Rev..

[14] Pérez-Pacheco E., Cauich-Cupul J.I., Valadez-González A., Herrera-Franco P.J. (2013). Effect of moisture absorption on the mechanical behavior of carbon fiber/epoxy matrix composites. J. Mater. Sci..

[15] Wang Z., Xian G., Zhao X.L. (2018). Effects of hydrothermal aging on carbon fibre/epoxy composites with different interfacial bonding strength. Constr. Build. Mater..

[16] Kumar B.G., Singh R.P., Nakamura T. (2002). Degradation of Carbon Fiber-reinforced Epoxy Composites by Ultraviolet Radiation and Condensation. J. Compos. Mater..

[17] Kumar B.G., Singh R.P., Nakamura T. (2002). Factors governing water absorption by composite matrices. Compos. Sci. Technol..

[18] Wang Z., Huang X., Xian G., Li H. (2016). Effects of Surface Treatment of Carbon Fiber : Tensile Property, Surface Characteristics, and Bonding to Epoxy. Polym. Compos..

[19] Jiang X., Kolstein H., Bijlaard F.S.K. (2012). Moisture diffusion and hygrothermal aging in pultruded fibre reinforced polymer composites of bridge decks. Mater. Des..

[20] Goertzen W.K., Kessler M.R. (2007). Dynamic mechanical analysis of carbon/epoxy composites for structural pipeline repair. Compos. Part B.

[21] Dao B., Hodgkin J., Krstina J., Mardel J., Tian W. (2006). Accelerated aging versus realistic aging in aerospace composite materials. II. Chemistry of thermal aging in a structural composite. J. Appl. Polym. Sci..

[22] Ramirez F.A., Carlsson L.A., Acha B.A. (2008). Evaluation of water degradation of vinylester and epoxy matrix composites by single fiber and composite tests. J. Mater. Sci..

[23] Guermazi N., Elleuch K., Ayedi H.F. (2010). The effect of time and aging temperature on structural and mechanical properties of pipeline coating. Mater. Des..

[24] Marouani S., Curtil L., Hamelin P. (2012). Ageing of carbon/epoxy and carbon/vinylester composites used in the reinforcement and/or the repair of civil engineering structures. Compos. Part B.

[25] Alessi S., Pitarresi G., Spadaro G. (2014). Effect of hydrothermal ageing on the thermal and delamination fracture behaviour of CFRP composites. Compos. Part B Eng..

[26] Kajorncheappunngam S., Gupta R.K., Gangarao H.V.S. (2002). Effect of Aging Environment on Degradation of Glass-Reinforced Epoxy. J. Compos. Constr..

[27] Stark W., Jaunich M., Mchugh J. (2015). Dynamic Mechanical Analysis (DMA) of epoxy carbon- fibre prepregs partially cured in a discontinued autoclave analogue process. Polym. Test..

[28] Sindhu K., Joseph K., Joseph J.M., Mathew T.V. (2007). Degradation studies of coir fiber/polyester and glass fiber/polyester composites under different conditions. J. Reinf. Plast. Compos..

[29] Zafar A., Bertocco F., Schjødt-Thomsen J., Rauhe J.C. (2012). Investigation of the long term effects of moisture on carbon fibre and epoxy matrix composites. Compos. Sci. Technol..

[30] Rocha I.B.C.M., Raijmaekers S., Nijssen R.P.L., vander Meer F.P., Sluys L.J. (2017). Hygrothermal ageing behaviour of a glass/epoxy composite used in wind turbine blades. Compos. Struct..

[31] Karbhari V.M., Xian G. (2009). Composites : Part B Hygrothermal effects on high V F pultruded unidirectional carbon/epoxy composites : Moisture uptake. Compos. Part B.

[32] Boubakri A., Haddar N., Elleuch K., Bienvenu Y. (2010). Impact of aging conditions on mechanical properties of thermoplastic polyurethane. Mater. Des..

[33] Zhang A.Y., Zhang D.X., Li D.H., Sun T., Xiao H.Y., Jia J. (2011). Tensile strength of hygrothermally conditioned carbon/epoxy composites with voids. Energy Procedia.

[34] Alzeebaree R., Çevik A., Nematollahi B., Sanjayan J. (2019). Mechanical properties and durability of unconfined and confined geopolymer concrete with fiber reinforced polymers exposed to sulfuric acid. Constr. Build. Mater..

[35] Hong B., Xian G. (2018). Ageing of a thermosetting polyurethane and its pultruded carbon fiber plates subjected to seawater immersion. Constr. Build. Mater..

[36] Kumar S., Sharma N., Ray B.C. Acidic degradation of FRP composites. Proceedings of the National Conference on Developments In Composites, National Institute of Technology.

[37] Amaro A.M., Reis P.N.B., Neto M.A., Louro C. (2013). Effects of alkaline and acid solutions on glass/epoxy composites. Polym. Degrad. Stab..

[38] Alamri H., Low I.M. (2012). Mechanical properties and water absorption behaviour of recycled cellulose fibre reinforced epoxy composites. Polym. Test..

[39] Boubakri A., Haddar N., Elleuch K., Bienvenu Y. (2011). Influence of thermal aging on tensile and creep behavior of thermoplastic polyurethane. Comptes Rendus Mec..

[40] Hayward D., Crane R.L., Pethrick R.A., Armstrong G.S., Banks W.M. (2004). Dielectric and mechanical studies of the durability of adhesively bonded CFRP structures subjected to aging in various solvents. Proc. Inst. Mech. Eng. Part L J. Mater. Des. Appl..

[41] Abanilla M.A., Karbhari V.M., Li Y. (2006). Interlaminar and intralaminar durability characterization of wet layup carbon/epoxy used in external strengthening. Compos. Part B.

[42] Wang Z., Zhao X., Xian G., Wu G., Raman R.K.S., Al-saadi S. (2017). Durability study on interlaminar shear behaviour of basalt-, glass- and carbon-fibre reinforced polymer (B/G/CFRP) bars in seawater sea sand concrete environment. Constr. Build. Mater..

[43] Wu G., Dong Z.-Q., Wang X., Zhu Y., Wu Z.-S. (2014). Prediction of Long-Term Performance and Durability of BFRP Bars under the Combined Effect of Sustained Load and Corrosive Solutions. Am. Soc. Civ. Eng..

[44] Chen Y., Davalos J.F., Ray I. (2007). Durability Prediction for GFRP Reinforcing Bars Using Short-Term Data of Accelerated Aging Tests. J. Compos. Constr..

[45] Chu W., Karbhari V.M. (2005). Effect of Water Sorption on Performance of Pultruded E-Glass/Vinylester Composites. J. Mater. Civ. Eng. ASCE.

[46] Yan L., Chouw N. (2015). Effect of water, seawater and alkaline solution ageing on mechanical properties of flax fabric/epoxy compos, ites used for civil engineering applications. Constr. Build. Mater..

[47] Qian X., Zhang Y.G., Wang X.F., Heng Y.J., Zhi J.H. (2016). Effect of carbon fiber surface functionality on the moisture absorption behavior of carbon fiber/epoxy resin composites. Surf. Interface Anal..

[48] Hong B., Xian G., Wang Z. (2018). Durability study opultruded carbon fiber reinforced polymer plates subjected to water immersion. Adv. Struct. Eng..

